# How to fight fake papers: a review on important information sources and steps towards solution of the problem

**DOI:** 10.1007/s00210-024-03272-8

**Published:** 2024-07-06

**Authors:** Jonathan Wittau, Roland Seifert

**Affiliations:** Institute of Pharmacology, Hannover Medical School, Carl-Neuberg-Straße 1, 30625 Hannover, Germany

**Keywords:** Paper mill, Fake paper, Scientific integrity, Manipulated data, Fabricated data, Research fraud

## Abstract

Scientific fake papers, containing manipulated or completely fabricated data, are a problem that has reached dramatic dimensions. Companies known as paper mills (or more bluntly as “criminal science publishing gangs”) produce and sell such fake papers on a large scale. The main drivers of the fake paper flood are the pressure in academic systems and (monetary) incentives to publish in respected scientific journals and sometimes the personal desire for increased “prestige.” Published fake papers cause substantial scientific, economic, and social damage. There are numerous information sources that deal with this topic from different points of view. This review aims to provide an overview of these information sources until June 2024. Much more original research with larger datasets is needed, for example on the extent and impact of the fake paper problem and especially on how to detect them, as many findings are based more on small datasets, anecdotal evidence, and assumptions. A long-term solution would be to overcome the mantra of publication metrics for evaluating scientists in academia.

## Many fake papers are produced by paper mills: a successful business model

Scientists who publish fake results are nothing new. A well-known example of this is a study that was intended to prove a link between the mumps-measles-rubella vaccination and autism (Wakefield et al. 1998), which turned out to be fraudulent (Godlee et al. 2011). In recent years, however, the problem has worsened dramatically. Falsified scientific papers are no longer isolated incidents but contaminate the scientific record on a large scale. In the scientific community, these papers have become known as fake papers. They contain intentionally manipulated data or are completely fictitious. Large numbers of fake papers are produced by so-called paper mills, also referred to more bluntly as criminal science publishing gangs (Byrne and Christopher 2020; Christopher 2021; Else and Van Noorden 2021; Sabel and Seifert 2021; Seifert 2021a; Van Noorden 2023a). Paper mills offer fake papers from various scientific disciplines and advertise them online. People can then buy authorship of a paper of their interest. The price depends on the authorship position and the impact factor of the journal in which the fake paper will be published later. Paper mills also handle the entire publication process (Abalkina 2023). The services of these paper mills make it easy for people with little (or even no) scientific experience to become authors of fake papers, which in many cases are done well enough to pass peer review and get published (Else and Van Noorden 2021). Key to the commercial success of paper mills are misguided scientists who misuse their expertise to falsify science and commercially exploit dysfunctional academic evaluation systems.

## How can I inform myself about fake papers? Unfortunately, much of the relevant information is not found in original peer-reviewed papers, but rather in other publication formats

This review provides an overview of important sources on the topic of fake papers and paper mills (Tables 1, 2, 3, 4, 5, 6, 7). These sources were categorized as follows: peer-reviewed original research (Table 1), reviews (Table 2), non-peer-reviewed preprints (Table 3), editorials (Table 4), journalistic perspective articles, news, views, and opinions (Table 5), blogs (Table 6), and further sources (Table 7). A related overview on this topic was published in 2021 (Van der Heyden 2021). Since then, there have been many new publications on this topic, so this review now provides an updated overview. However, this review does not claim to be complete and is limited to a deadline of June 2024.

**Table 1:** Original peer-reviewed papers dealing with “ truthl fake paper research”

Bibliography	Content of the article
Abalkina A (2023) Publication and collaboration anomalies in academic papers originating from a paper mill: Evidence from a Russia-based paper mill. Learned Publishing 36:689–702	Insights into the business practices of a Russian paper mill. A total of 451 papers possibly linked to this paper mill were identified by searching for publications for which the paper mill had previously offered authorships for sale. Furthermore, other anomalies were found in these papers that could indicate the involvement of this paper mill
Abalkina A, Bishop D (2023). Paper mills: a novel form of publishing malpractice affecting psychology. Meta-Psychology 7	Six publications of the *Journal of Community Psychology* are suspected of originating from a paper mill. The suspicion is based on several features of these publications. This includes the fact that the different reviewers of these papers always submitted their reports at the same time, which would otherwise never be the case in this journal
Bucci EM (2018) Automatic detection of image manipulations in the biomedical literature. Cell Death Dis. 9(3):400	Images in scientific publications were automatically scanned for signs of image manipulation. Image manipulation was found in 6% of all articles examined. In papers containing images of gel electrophoresis, the proportion of manipulated images was even 22%
Byrne JA, Labbé C (2017) Striking similarities between publications from China describing single gene knockdown experiments in human cancer cell lines. Scientometrics 110:1471–1493	Identification of 48 very similar publications describing the effects of single gene knockdowns in human cancer cell lines. It is suspected that in some cases the authors of these publications did not conduct the experiments themselves. Instead, they may have used data and figures that originate from companies without indicating this. The validity of the results of these studies is generally questioned
Candal-Pedreira C, Ruano-Ravina A, Fernández E, Ramos J, Campos-Varela I, Pérez-Ríos M (2020) Does retraction after misconduct have an impact on citations? A pre-post study. BMJ Glob Health 5(11):e003719	Retractions do not prevent fraudulent scientific publications from being cited. This is shown by an analysis of 304 original articles and reviews that were retracted due to misconduct
Candal-Pedreira C, Ross JS, Ruano-Ravina A, Egilman DS, Fernández E, Pérez-Ríos M (2022) Retracted papers originating from paper mills: cross sectional study. BMJ 379:e071517	Analysis of some general characteristics of 1182 publications that were retracted because they originated from paper mills
Dadkhah M, Oermann MH, Hegedüs M, Raman R, Dávid LD (2023) Detection of fake papers in the era of artificial intelligence. Diagnosis. 10.1515/dx-2023–0090	Applying machine learning to suggested features of fake papers to create decision trees that can be used to determine whether a paper might be fake
Gopalakrishna G, Ter Riet G, Vink G, Stoop I, Wicherts JM, Bouter LM (2022) Prevalence of questionable research practices, research misconduct and their potential explanatory factors: A survey among academic researchers in The Netherlands. PLoS One 17(2):e0263023	Survey on scientific integrity among academics of all disciplines in the Netherlands. Based on the responses of the 6813 respondents, the prevalence of data fabrication and data falsification is estimated to be 4.2% and 4.3%, respectively. The prevalence is even higher for other scientifically questionable practices. According to the survey, publication pressure is the most important reason for this
Labbé C, Grima N, Gautier T, Favier B, Byrne JA (2019) Semi-automated fact-checking of nucleotide sequence reagents in biomedical research publications: The Seek & Blastn tool. PloS one 14(3)	Development and testing of a tool for semi-automatic identification of papers with incorrect nucleotide sequences. The authors recommend this tool for detecting fake papers
Oksvold MP (2016) Incidence of Data Duplications in a Randomly Selected Pool of Life Science Publications. Sci Eng Ethics 22(2):487–96	A total of 120 articles from three different cancer journals were screened for figure duplication. Around a quarter of the analyzed publications contained such duplications. It was not possible to determine whether these were accidental or intentional
Park Y, West RA, Pathmendra P, Favier B, Stoeger T, Capes-Davis A, Cabanac G, Labbé C, Byrne JA (2022) Identification of human gene research articles with wrongly identified nucleotide sequences. Life science alliance 5(4):e202101203	Checking the nucleotide sequences in more than 3400 research articles. Identification of 712 articles published in 78 journals containing a total of more than 1500 incorrect sequences. The authors suspect that a part of these articles originate from paper mills
Pathmendra P, Park Y, Enguita FJ, Byrne JA (2024) Verification of nucleotide sequence reagent identities in original publications in high impact factor cancer research journals. Naunyn Schmiedebergs Arch Pharmacol	Verification of the nucleotide sequences of 552 publications from two different cancer journals with a high impact factor. The prevalence of incorrectly identified nucleotide sequences increased over the years, reaching up to 38% and 40% respectively in 2020. The authors suspect that attacks by paper mills on journals with a high impact factor are one possible explanation. They conclude that a journal’s high impact factor is no guarantee of high research quality in the articles
Wittau J, Celik S, Kacprowski T, Deserno TM (2023) Fake paper identification in the pool of withdrawn and rejected manuscripts submitted to Naunyn– Schmiedeberg’s Archives of Pharmacology. Naunyn–Schmiedeberg’s Arch Pharmacol 397(4):2171–2181	Identification of publications that have been submitted to different journals, but for which the listed authors have been exchanged in between. The authors suspect that paper mills are involved in such manuscripts and suggest this approach for detecting fake papers. The method was tested on 2056 withdrawn or rejected submissions from Naunyn–Schmiedeberg’s Archives of Pharmacology, and ten potential fake papers were discovered
Wittau J, Seifert R (2023) Metadata analysis of retracted fake papers in Naunyn- Schmiedeberg’s Archives of Pharmacology. Naunyn–Schmiedeberg’s Arch Pharmacol 397(6):3995-4011	Metadata analysis of 12 proven fake papers published in Naunyn–Schmiedeberg’s Archives of Pharmacology. The comparison with 733 reference articles from the same journal shows that fake papers hardly differ from other articles at the metadata level. Furthermore, it is shown that several features proposed in other articles for the identification of fake papers are either not usable or only of limited use based on the analyzed dataset

**Table 2:** Reviews dealing with fake papers

Bibliography	Summary
Byrne JA, Grima N, Capes-Davis A, Labbé C (2019) The Possibility of Systematic Research Fraud Targeting Under-Studied Human Genes: Causes, Consequences, and Potential Solutions. Biomarker insights 14:1177271919829162	It is argued that the existence of thousands of under-researched human genes that could be studied in the context of various diseases, combined with an increase of specialized journals that sometimes publish such research without adequate peer review, partly due to a lack of experts, favors a situation in which paper mills can easily produce large quantities of fake papers following the same template. The authors report that they have identified numerous very similar publications that most likely originate from Paper Mills and were created using templates. They warn that such fake papers could misdirect future research, particularly in cancer biomarkers. Furthermore, some features are discussed that could help to detect such cases
Van der Heyden MAG (2021) The 1-h fraud detection challenge. Naunyn–Schmiedeberg’s Arch Pharmacol 394(8):1633–1640	Presentation of a workflow for detecting image manipulation in scientific publications and a collection of literature on the topic of fake papers

**Table 3:** Non-peer-reviewed preprints dealing with fake papers

Bibliography	Summary
Cabanac G, Labbé C, Magazinov A (2021) Tortured phrases: a dubious writing style emerging in science. Evidence of Critical Issues Affecting Established Journals. ArXiv:2107.06751. 10.48550/arXiv.2107.06751	Identification of publications that contain strange expressions instead of established terms. These expressions are probably the result of the papers being created or rewritten using artificial intelligence. The authors suspect that some of these publications originate from paper mills
Sabel BA, Knaack E, Gigerenzer G, Bilc M (2023) Fake publications in biomedical science: red-flagging method indicates mass production. MedRxiv 2023.05.06.23289563	The use of non-institutional email addresses, the absence of international co-authors, and whether the authors’ institutions are hospitals are used as features to identify potential fake papers. The authors estimate the proportion of potential fake papers at 11% in 2020
Qi C, Zhang J, Luo P (2020). Emerging Concern of Scientific Fraud: Deep Learning and Image Manipulation. BioRxiv 2020.11.24.395319	Artificial intelligence is used to generate western blot images. Experts who were shown these images were unable to distinguish the real images from the generated images

**Table 4:** Editorials dealing with fake papers

Bibliography	Summary
Frederickson RM, Herzog RW (2021) Keeping Them Honest: Fighting Fraud in Academic Publishing. Mol Ther 29(3):889–890	It is described how *Molecular Therapy* is trying to defend itself against fake papers. Part of their approach is to uniquely identify authors by requiring full institutional contact information, ORCID IDs, institutional email addresses, and institutional phone numbers, which are then verified by the journal. If fraud is detected in a paper, the authors will be banned from submitting further papers to the journal. Additionally, their affiliated institution will be notified and, if necessary, the entire institution may be excluded from submitting future research
Hackett R, Kelly S (2020) Publishing ethics in the era of paper mills. Biol Open 9(10):bio056556	It is explained what *Biology Open* intends to do against fake papers. For example, a Publishing Ethics Coordinator was hired to investigate suspected cases. In addition, suspicious cases that have already been published will be publicly flagged and retracted if necessary
Heck S, Bianchini F, Souren NY, Wilhelm C, Ohl Y, Plass C (2021) Fake data, paper mills, and their authors: The International Journal of Cancer reacts to this threat to scientific integrity. Int. J. Cancer 149:492–493	How the *International Journal of Cancer* attempts to identify and reject submissions with integrity issues, especially those from paper mills. The journal estimates that up to 10% of submitted manuscripts show signs of potential paper mill involvement, and up to 5% of submitted manuscripts have serious integrity problems
Miyakawa T (2020) No raw data, no science: another possible source of the reproducibility crisis. Mol Brain 13(1):24	An editor requested original data for 41 conspicuous submissions. In 21 cases, the authors withdrew their papers without providing original data. In 19 cases, the authors provided insufficient original data. The editor suspects that some of these papers have been fabricated and appeals to journals to make an effort to publish the original data when publishing a paper
Sabel BA, Seifert R (2021) How criminal science publishing gangs damage the genesis of knowledge and technology—a call to action to restore trust. Naunyn- Schmiedeberg’s Arch Pharmacol 394:2147–2151	Two editors-in-chief of scientific journals appeal to tackle the problem of fake papers and suggest measures that journals could take
Seifert R (2021) How Naunyn–Schmiedeberg’s Archives of Pharmacology deals with fraudulent papers from paper mills. Naunyn–Schmiedeberg’s Arch Pharmacol 394(3):431–436	How *Naunyn–Schmiedeberg’s Archives of Pharmacology* deals with fake papers and what steps the journal is taking to combat the problem. A list of features that the editors noticed in the fake papers is provided

**Table 5:** Other article formats (including journalistic articles) dealing with fake papers

Bibliography	Content of the article
Perspectives	
Bik EM, Casadevall A, Fang FC (2016) The Prevalence of Inappropriate Image Duplication in Biomedical Research Publications. Mbio 7(3):e00809-16	Images in more than 20,000 scientific publications from 40 different journals were manually checked for problematic image duplication. A total of 3.8% of these publications contained at least one problematic figure, and most of these cases cannot be explained by unintentional mistakes. The prevalence of problematic images varied depending on the journal and country of origin of the publications
Byrne JA, Christopher J (2020) Digital magic, or the dark arts of the twenty-first century— how can journals and peer reviewers detect manuscripts and publications from paper mills? FEBS Lett 594:583–589	Summary of the business model of paper mills. Several features of fake papers that may occur as a result of mass production by paper mills are proposed. Suggestions are made on how journals can screen for these features
Christopher J (2018) Systematic fabrication of scientific images revealed. FEBS letters 592(18):3027–3029	FEBS Press journals have received submissions containing manipulated images of western blots. The background patterns of the Western blots were sometimes identical in submissions from different authors, suggesting a common source for the manipulation of these images
Christopher J (2021) The raw truth about paper mills. FEBS Lett 595:1751–1757	Recommendation to request original data if there is any suspicion that a submitted work could be fake. Paper mills would not be able to provide original data or would fabricate it. Examples of manipulated original data are shown
Opinions	
Byrne J (2019) We need to talk about systematic fraud. Nature 566(7742):9	Call for less ignorance of systematic research fraud
Christos P (2024) Guest Post – Making Sense of Retractions and Tackling Research Misconduct. The Scholarly Kitchen. https://scholarlykitchen.sspnet.org/2024/04/18/guest-post-making-sense-of-retractions-and-tackling-research-misconduct/?informz=1&nbd=dbf10c0a-9e11-4bf3-af3b-d2e383056ded&nbd_source=informz. Accessed May 6, 2024	It is argued that the increase in retractions of scientific papers in recent years is not due to a general increase in scientific misconduct. Instead, it is claimed that the increase can only be attributed to individual players, namely individual journals with high retraction rates, individual authors with numerous retractions, and China, which is a major contributor to global retractions. Some statistics on retractions in recent years are shown
Korte SM, van der Heyden MA (2017) Preventing Publication of Falsified and Fabricated Data: Roles of Scientists, Editors, Reviewers, and Readers. J Cardiovasc Pharmacol 69(2):65–70	Some actions against research fraud are suggested. This includes implementing pre-review screening and post-publication peer review, training scientists in detecting and dealing with fraud, and creating awareness and open discussion within the institution
Wang L, Zhou L, Yang W, Yu R (2022) Deepfakes: A new threat to image fabrication in scientific publications? Patterns (N Y) 3(5):100509	The authors demonstrate that it is possible to create deep fakes of scientific images and warn that these are difficult to identify
News and views	
Else H (2019) What universities can learn from one of science’s biggest frauds. Nature 570(7761):287–288	Scientists criticize deficiencies in institutional investigations of research misconduct. They suggest involving external experts, using standardized checklists, and focusing more on correcting or retracting papers to ensure the integrity of the scientific literature
Else H, Van Noorden R (2021) The fight against fake-paper factories that churn out sham science. Nature 591:516–519	Report on different paper mill cases, their origins, their detection and retraction, as well as the journals’ approaches to solving the problem
Else H (2022) Paper-mill detector put to the test in push to stamp out fake science. Nature 612(7940):386–387	Publishers are testing automated tools to detect potential fake papers. These tools are developed in collaboration with several publishers. In the long term, these tools are intended to recognize paper mill articles by numerous features, including image manipulation and the simultaneous submission of an article to several journals
Hvistendahl M (2013) China’s publication bazaar. Science 342(6162):1035–9	An investigation has uncovered the trade of scientific publications and authorships in China. Insights into the business practices of paper mills are provided and possible causes of the problem are discussed
Jones N (2024) How journals are fighting back against a wave of questionable images. Nature 626(8000):697–698	Journals integrate software that can recognize problematic images into their pre-publication checks. Although this software helps, it is not able to recognize complex image manipulations
Kupferschmidt K (2018) Researcher at the center of an epic fraud remains an enigma to those who exposed him. Science 80	Report on a scientist who fabricated numerous clinical trials on how to prevent bone fractures. These studies have distorted the results of meta-studies, may have influenced medical guidelines, and initiated real clinical trials
Liverpool L (2023) AI intensifies fight against ‘paper mills’ that churn out fake research. Nature 618(7964):222–223	Researchers warn that paper mills are using generative AI to create text and images for fake papers. Furthermore, publishers are considering how an exchange of information between publishers could help against paper mills
Mallapaty S (2024) China conducts first nationwide review of retractions and research misconduct. Nature 626(8000):700–701	The Chinese Ministry of Education has called on Chinese universities and researchers to declare retracted papers originating from them. They have to explain why these papers have been retracted and, in cases of misconduct, universities must initiate investigations
Marcus A (2018) A scientist’s fraudulent studies put patients at risk. Science 362(6413):394	A case in which a scientist is accused of falsifying data in more than 90 publications. As a result, medical guidelines in the UK recommended the use of hydroxyethyl starch solutions, which would not have been the state of the art without the fabricated studies
Piller C. Blots on a field? (2022) Science 377(6604):358–363	Numerous articles in Alzheimer’s research are suspected of having been falsified. This includes one of the most highly regarded Alzheimer’s studies
Shen H (2020) Meet this super-spotter of duplicated images in science papers. Nature 581(7807):132–136	Introduction of a fake paper detective. The commitment of such people is responsible for the detection of many fake papers
Van Noorden R (2021) Hundreds of gibberish papers still lurk in the scientific literature. Nature 594(7862):160–161	Researchers have discovered 243 papers created by software that generates random text that is easily recognizable as nonsense
Van Noorden R (2022) Journals adopt AI to spot duplicated images in manuscripts. Nature 601(7891):14–15	Publishers are developing and testing tools to automatically detect image manipulation in submitted articles
Van Noorden R (2023) Medicine is plagued by untrustworthy clinical trials. How many studies are faked or lawed? Nature 619(7970):454–458	Some researchers state that in their experience 20–30% of all medical RCTs have integrity problems, and they see intentional fabrication as one of the causes. Cases of fake RCTs are mentioned and the potential risk for patients is pointed out. The treatment of untrustworthy studies in reviews is also discussed
Van Noorden R (2023) How big is science’s fake-paper problem? Nature 623(7987):466–467	Report on three different estimates of the proportion of fake papers, ranging from 1.5 to 11%
Van Noorden R (2023) More than 10,000 research papers were retracted in 2023—a new record. Nature 624(7992):479–481	Statistics are shown on how many publications have been retracted in recent years. In 2023, there was a provisional high of 10,000 retractions, which corresponds to a retraction rate of 0.2%. Furthermore, the countries with the most retractions are listed

**Table 6:** Science blogs dealing with fake papers

Bibliography	Summary
For Better Science https://forbetterscience.com/	Blog on integrity and ethics in research, with numerous articles on fake papers and paper mills. Here, some fake paper detectives share their concerns about publications in which they have detected fraud
PubPeer https://pubpeer.com/	Website that allows public comments on scientific publications in the sense of a post-publication peer review
Retraction Watch https://retractionwatch.com/	Blog that reports on retractions of scientific publications. Furthermore, Retraction Watch is a database that lists more than 45,000 retractions
Science Integrity Digest https://scienceintegritydigest.com/	Blog about research integrity, with many articles showing fraud in fake papers

**Table 7:** Further information sources on fake papers

Bibliography	Summary
COPE & STM (2022) Paper Mills — Research report from COPE & STM — English. https://publicationethics.org/node/55256 10.24318/jtbG8IHL Accessed October 22, 2023	Overview of the problem of paper mills and an analysis of its scale
STM — International Association of STM Publishers (November 28, 2022) Image alteration and duplication in scientific publications — Module 1. [video]. YouTube: https://www.youtube.com/watch?v=-taHMZgh-9Q Accessed December 16, 2023	Video tutorial on common scientific image fraud and how to detect it
STM — International Association of STM Publishers (May 22, 2023) Image alteration and duplication in scientific publications | Module 2. [video]. YouTube: https://www.youtube.com/watch?v=UddQodWU__8 Accessed December 16, 2023	Video tutorial on common scientific image fraud and how to detect it

## Why people publish fake papers: from desperate doctors and established scientists seeking extra prestige

In the academic world, the number of publications is used as one benchmark to evaluate scientists (Abbott et al. 2010; Kumar et al. 2009; Rawat and Meena 2014; Walker et al. 2010). Particularly in China, but not exclusively, there are strong incentives to publish scientific papers, especially in high-impact factor journals. These incentives include jobs, tenure, promotion, grants, and even personal financial rewards (Abritis and Mccook 2017; Hvistendahl 2013; Quan et al. 2017; Tian et al. 2016; Zhang and Wang 2024). Without publications, promotion is often not possible in China, not even for medical doctors (Hvistendahl 2013; Tian et al. 2016). However, a lack of time and resources can make it difficult for many scientists to publish enough papers. And of course, for a medical doctor with full patient responsibility, the conduct of high-quality research is almost impossible. Unfortunately, under these circumstances, some people are tempted to publish fake papers. Data from real studies are then falsified, or entire studies are freely invented (Byrne and Christopher 2020; Else and Van Noorden 2021; Gopalakrishna et al. 2022; Miyakawa 2020; Seifert 2021a). The fact that many “authors” of fake papers use the expensive services of paper mills to do this shows two aspects: First, the pressure or incentive to publish is apparently so high that they accept the high costs of a paper mill (Abalkina 2023; Hvistendahl 2013). Second, these people are apparently so inexperienced with scientific publications or have so little time that they do not produce the fake papers themselves. It is also possible that the paper mills claim to use real research data (Hvistendahl 2013) and that paper mill customers therefore assume that the papers they buy are based on real studies.

Apart from the professional and financial benefits, there may be other reasons why someone would publish a fake paper. Diederik Stapel is a scientist who has been proven to have fabricated data in 58 of his papers (Palus 2015). He continued to fabricate study data long after his professional career was already considered glorious. He described his reasons for doing so as complex but also stated ambition and the desire to present excellent data in his papers (Bhattacharjee 2013). Eric Poehlman (Dahlberg and Mahler 2006) and Hwang Woo-suk (Wade and Sang-Hun 2006) are other well-known examples of scientists who have faked some of their work despite their advanced careers. These two also do not fit the narrative described above of desperate scientists or doctors who see no other option for their careers than to hire a paper mill. Hwang Woo-suk, for example, claimed the prestige of having cloned human embryonic stem cells (Wade and Sang-Hun 2006), so his ambitions presumably went beyond simply “surviving” in the scientific business. Thus, it seems that in individual cases, scientific prestige is also a motivation to fake results. In such cases, paper mills are not involved in the fake paper production.

## How big is the fake paper problem? Estimates vary greatly and reflect methodological difficulties

It is hard to estimate the proportion of fake papers in the scientific record. Currently, different numbers are circulating. Two journals that had noticed submissions from paper mills estimated the proportion of such submissions at about 5–10% and 5%, respectively (Heck et al. 2021; Seifert 2021a). However, such estimates of individual journals are not representative, as paper mills attack certain journals more frequently (COPE & STM 2022). The proportion of submitted fake papers varied widely across disciplines and journals, with rates up to 46% (COPE & STM 2022). Another study found that about 0.01% of all scientific papers published in 2019 were retracted because they originated from a paper mill (Candal-Pedreira et al. 2022). Since by far not all fake papers are detected and retracted, it can be assumed that this proportion underestimates the actual amount of published fake papers. A news article reported that in 2023, more than 10,000 publications were retracted globally and across all disciplines, most of them for reasons indicating that they were fake papers. The article also shows a gradual increase in retraction rates over the last 20 years, which exceeded 0.2% in 2023 (Van Noorden 2023a). This increase may be due to both an increasing number of published fake papers and greater awareness of the problem. Another article reported on an unpublished study that found strong textual similarities with already known fake papers in 1.5 to 2% of all papers published in 2022. The suspicion was expressed that many of these papers could be fake papers too, but it was also pointed out that the actual proportion of fake papers cannot be determined without individual examination of these suspected cases (Van Noorden 2023b). In a screening of around 20,000 articles published in various journals between 1995 and 2014, problematic images were found in 3.8% of cases (Bik et al. 2016). Another study of more than 1300 randomly selected open-access articles published by PubMed Central in January 2014 found problematic images in 6% of cases (Bucci 2018). Many of the problematic images in these studies were intentionally fabricated, leading to suspicions that the publications in question might be fake papers. One study even estimated that 28.8% of all biomedical papers published in 2020 were potential fake papers. However, one of the three criteria used to classify publications as suspicious in this study was the use of non-institutional email addresses (Sabel et al. 2023). A different study concluded that this criterion is not suitable for identifying fake papers (Wittau and Seifert 2023), so the 28.8% is most likely a considerable overestimate. The same applies to another version of this study, in which the authors reduced their estimate to 11% but retained the methodology (Sabel et al. 2023). In a study focusing on rejected and withdrawn papers in *Naunyn-Schmiedebergs Arch Pharmacol* that were finally published elsewhere, the percentage of verified fake papers was below 1% (Wittau et al. 2023).

## The fairy tale that high-impact journals are not affected by fake papers

In the scientific community, it is often assumed that fake papers are only a problem of low-medium scientific journals, but this is simply not true. A recent systematic analysis revealed that the problem is very pertinent in high-impact factor oncology journals (Pathmendra et al. 2024). These links list articles that have been retracted by Nature (https://www.nature.com/nature/articles?type=retraction; accessed June 17, 2024) and Science (https://pubmed.ncbi.nlm.nih.gov/?term=%28%22Science+%28New+York%2C+N.Y.%29%22%5BJournal%5D%29+AND+%28%22Retraction+of+Publication%22%5Bpt%5D%29&size=200; accessed June 17, 2024). Many of the retractions occurred due to honest errors, but fraud was also a recurring reason. These cases show that even the most respected and prestigious journals, having access to the best reviewers globally, can be effectively fooled by fake paper authors. In the case of this top category of journals, the desire of editors to publish the most impactful, sensational, and attention-drawing papers may, at least in some cases, impede the objective assessment of papers.

## The damage that fake papers produce in science

As the extent of the fake paper problem is not clear, there are no studies to date that capture its impact. This section is therefore speculative and only lists a few probable consequences and examples.

Contamination of the scientific record by fake papers undermines the reliability of scientific studies. This particularly affects scientists who base their studies on the published results of other scientists. Fake papers likely contribute to the reproducibility crisis (Miyakawa 2020) and therefore lead to a waste of resources in science and industry. A well-known example of this can be found in Alzheimer’s research. One of the most highly regarded Alzheimer’s studies (Lesné et al. 2006), which has helped shape the direction of Alzheimer’s research and treatment development in recent years, has been suspected of being falsified for some time. If this suspicion is confirmed, this paper could have contributed to a waste of millions or even billions of dollars and the efforts of numerous people (Piller 2022). Another famous example can be found in research on room-temperature superconductors. Three papers that had caused a sensation in this research area have been recently retracted (Dasenbrock-Gammon et al. 2023; Durkee et al. 2021; Snider et al. 2020). The reasons for the retractions were doubts about the integrity of the data (Castelvecchi 2023; Garisto 2023). There are no numbers on how much money and time other scientists have wasted because of these studies. However, since the three studies together were cited very often (Dasenbrock-Gammon et al. 2023; Durkee et al. 2021; Snider et al. 2020) and there were various attempts to reproduce the results (Castelvecchi 2023; Garisto 2023), it can be assumed that they led to a substantial waste of resources. Of course, these are two extreme examples of the consequences scientific fraud can have. Most fake papers will presumably be much less influential. It is also conceivable that fake papers not only lead to unjustified research efforts in a particular area but also discourage honest scientists from conducting research in areas where results already appear to be available.

In addition to the harm to science and the economy, fake papers could also have a direct impact on society. If scientific studies lose credibility in the public eye, people who deny scientific evidence may feel confirmed, and public discourse on important social issues such as climate change or vaccination may be damaged (Hopf et al. 2019).

Medical fake papers could even lead to negative consequences for patients (Van Noorden 2023c). One example of this is a case in which a researcher is alleged to have fabricated data in over 90 publications, many of which contained a positive assessment of the risk–benefit ratio of infusions containing hydroxyethyl starch solutions. These publications influenced, for example, the guidelines of several medical societies in the UK. After the retraction of these publications, the data from the remaining studies indicated a worse risk–benefit ratio of hydroxyethyl starch solutions. At that time, however, many patients worldwide had already been treated with them under a different assumption (Marcus 2018). In another case, a researcher is believed to have faked numerous clinical trials investigating the benefits of various drugs and supplements to prevent bone fractures. Consequently, the results of meta-studies were distorted, a Japanese guideline may have been influenced, and real clinical trials were initiated based on the fabricated results (Else 2019; Kupferschmidt 2018). Another example is that some questionable studies have claimed that the drug ivermectin is effective against COVID-19. These studies were included in some meta-analyses, which came to incorrect conclusions regarding the efficacy of this drug against COVID-19 (Lawrence et al. 2021). Further meta-analyses showed that ivermectin is probably of no use in the prevention and treatment of COVID-19 infections (Popp et al. 2022). Nevertheless, many patients were treated with the drug because of the previous results (Mega 2020). The questionable studies were not explicitly stated to be intentional fakes. Instead, a more cautious wording was used, for example, stating that these studies were problematic, that there were several irregularities in the data, and that there were concerns about misconduct and a lack of research integrity (Lawrence et al. 2021; Popp et al. 2022).

## How fake papers can be detected: there is no simple single method

It is necessary to identify fake papers to retract them and to prevent their publication. Unfortunately, this is not easy. There are currently different approaches to detecting fake papers, all of which have their limitations. These approaches can be broadly divided into those that identify problems in the content of papers and those that focus on non-content-related anomalies. Some of them are time-consuming, while others can be used automatically on a large scale.

### Content-related approaches to detect fake papers

An easy way to weed out some potential fake papers at an early stage is to ask for original data at the time of submission. Many fake authors seem unprepared for this request and cannot provide original data (Miyakawa 2020; Seifert 2021a). A much more complicated approach is to identify manipulated images (Byrne and Christopher 2020; Seifert 2021a). Identifying such manipulations by hand is time-consuming and requires substantial skill. Van der Heyden (2021) and two videos by STM (STM 2022, 2023) describe possible procedures for recognizing image manipulation. Software is now being tested that can be used to automatically search for problematic images on a larger scale (Bucci 2018; Van Noorden 2022). Another indication of possible fake papers is errors in nucleotide sequences, which can be detected semi-automatically (Park et al. 2022).

### Non-content-related approaches to detect fake papers

In addition to missing, incorrect, or manipulated data, there are other signs that a paper may be fraudulent. One approach focuses on peer reviews. It seems to happen that paper mills successfully pose as reviewers. This way, they can provide positive reviews of their own papers. Identical reviews that come from different reviewers may indicate fake reviewers. It may also be conspicuous if different reviewers submit their reviews for the same paper at the same time. All papers handled by these reviewers should then be checked again in detail to see whether they are fake papers (Abalkina and Bishop 2023; Day 2022). *Naunyn-Schmiedebergs Arch Pharmacol* therefore ignores author suggestions for referees, at least reducing the probability of fake reviews.

Another method focuses on the writing style of papers. To reduce the effort of writing a fake paper, some people use artificial intelligence to either create new text or rewrite text from other publications and claim it as their own. Sometimes, this results in very strange expressions, which have been called “tortured phrases.” Systematically scanning for such phrases can therefore identify potential fake papers (Cabanac et al. 2021). Even without the occurrence of “tortured phrases,” papers written by artificial intelligence can be identified automatically and with high accuracy by the writing style (Desaire et al. 2023a, 2023b). *Naunyn-Schmiedebergs Arch Pharmacol* uses software for the detection of language manipulation and rejects all papers with signs of attempted plagiarism. It is also possible to compare papers automatically with already proven fake papers. A strong textual similarity can indicate that the examined papers originate from the same paper mill (Van Noorden 2023b). A further approach is to scan for papers that have been submitted to different journals but with different author lists. The background is that paper mills exploit the fact that journals are unaware of submissions to other journals. This allows them to change the customers for the purchased authorships while submitting papers to different journals (Wittau et al. 2023). It is also possible to look for papers that have previously been publicly offered for sale by a paper mill (Abalkina 2023). Finally, there are several anomalies and metadata-based features that may serve as additional indicators of fake papers (Abalkina 2023; Dadkhah et al. 2023; Sabel et al. 2023; Seifert 2021a; Wittau and Seifert 2023).

To date, there is no perfect method for detecting fake papers. The methods presented here are unfortunately only applicable under certain conditions. For this reason, tools are now being developed that combine many different methods. However, it is not publicly stated how exactly these tools are supposed to identify fake papers (Else 2022). Although paper mills will likely adapt to methods of detecting fake papers, the use of such tools will hopefully make their work more difficult in the future.

## Training scientists, authors, editors, and reviewers to detect fake papers is crucial

The most important step in keeping the scientific record clean of fake papers is to prevent them from getting published. Ideally, fake papers are already identified in a pre-review screening by tools or experts for detecting fakes, thus relieving the burden on scientific reviewers. Training opportunities for reviewers to recognize fraud would also be helpful (Korte and van der Heyden 2017). Retracting already published fake papers is also a very important step, but unfortunately less effective, because it does not prevent the fake papers from being cited in the meantime, and the citing does not even stop after the retraction (Candal-Pedreira et al. 2020; Wittau and Seifert 2023). Publishing notes of concern is a good way to be transparent about problems in a publication until a detailed investigation of a potential fake paper case has been completed (Seifert 2021b).

## Retractions should be viewed as positive tools to restore trust

Handling fake papers requires a huge effort and commitment on the part of the journals. *Molecular Therapy, Biology Open*, the *International Journal of Cancer*, and *Naunyn–Schmiedeberg’s Archives of Pharmacology* are examples of journals that openly address the problem of fakes within their own submissions and publications and take care of it (Frederickson and Herzog 2021; Hackett and Kelly 2020; Heck et al. 2021; Seifert 2021a). All these journals have already retracted fake papers. Unfortunately, there are also several respected journals that disregard evidence that several of their publications are fake and do not take appropriate action (Wittau et al. 2023). But the longer they do not act, the more severely will their reputation be damaged in the long run. There are also bad cases where papers are retracted silently and without any explanation. Specifically, all evidence of the paper’s existence is removed from the journal’s website (Teixeira da Silva and Daly 2024). We can only encourage all journals to publish retraction notes proactively, even if there will be many of those. From the experience with *Naunyn-Schmiedebergs Arch Pharmacol*, we can tell that such measures are appreciated by the scientific community and lead to an increase in honest high-quality submissions. But if journals follow the policy of ignoring the fake paper problem and fail to publish retraction notes although suspicious papers are published in this journal, the reputation of the respective journal will be damaged in the scientific community by spreading the word informally among scientists.

## Software tools provided by publishers to fight fake papers

Publishers should be responsible for ensuring that editorial boards can maintain the scientific integrity of their journals. They should support them with tools, training, and dedicated research integrity resources to which editors and reviewers can turn when encountering suspicious papers. Such integrity tools could also be made contactable for readers. In this way, the readership could be actively involved in post-publication peer review (Korte and van der Heyden 2017). Leading scientific journal publishers such as Springer Nature are aware of the urgency to develop software tools to detect fake papers more effectively. For example, the software tools Geppetto und SnappShot will support editors and referees alike to detect more easily text sophisticated manipulations and image manipulations, respectively (https://group.springernature.com/fr/group/media/press-releases/new-research-integrity-tools-using-ai/27200740; last accessed June 17, 2024). Such long-awaited software tools will constitute a major support for editors and reviewers to catch fake papers in the future that would have smoothly passed review despite diligent efforts. Internal author-flagging software systems in editorial systems will also be helpful to identify suspicious authors and catch fake papers.

Even more effective would be blacklists of suspicious authors shared across different publishers and journals because switching between different journals and publishers is an effective strategy for fake authors not being caught (immediately). Currently, fake paper authors aggressively abuse the absence of flagging systems to their advantage because they know that there are high barriers to the implementation of such data bases including reasons of discrimination, fairness, personal data protection, and political correctness.

## Substantial cultural changes at different levels are needed to weed out fake papers

To effectively combat fake papers, editors and reviewers need to become more aware of the problem and more willing to act against it. There is no point in identifying fake papers if they are not subsequently retracted or at least flagged by the journals (Wittau et al. 2023). We noted that despite the fake paper problem in *Naunyn-Schmiedebergs Arch Pharmacol*, the number of submissions and published honest papers has increased substantially over the past years in this journal. Apparently, honest authors value the approach of the journal to retract every single fake paper that the Editor-in-Chief becomes aware of. Thus, retraction of every fake paper pays off in many respects, for the scientific community, the honest authors, and the reputation of the affected journal. Fortunately, there are journals that share this perspective. At a recent symposium, the editors-in-chief of several journals met to discuss different strategies against fake papers and declared their intention to work together on this issue (Benyó et al. 2024).

Unfortunately, paper mills will not give up their business model as long as it remains financially lucrative and as long as there are well-paying customers. Presumably, they will continually adapt their methods to make their papers undetectable by current fake paper scanning methods (Abalkina 2023). To solve the fake paper problem in the long term, it will be necessary to address the cause of this problem: academic systems that put scientists under too much pressure to publish and reward them based on publication metrics. Revising academic systems globally would not only help against fake papers. Many (presumably) honestly conducted studies cannot be reproduced either (Baker 2016; Begley and Ellis 2012). In two surveys of scientists, most respondents stated that pressure to publish would be one of the reasons for this. The surveys did not explain further how this should be connected (Baker 2016; Samuel and König-Ries 2021). Perhaps the connection is that the thoroughness with which research is planned, conducted, and analyzed suffers from the need to produce many publications in a short period of time (Diaba-Nuhoho and Amponsah-Offeh 2021). Furthermore, the practice of evaluating scientists based on their publication metrics was cited as detrimental to scientific innovation. According to this theory, many scientists would respond by focusing on established research areas, as they feel they are more likely to get publications and citations this way. Novel and unconventional ideas would therefore be less likely to be pursued (Bhattacharya and Packalen 2020; Foster et al. 2015). The final example to be mentioned here is that focusing too much on a professor’s publications can lead to professors being hired at universities with insufficient consideration of their teaching skills (Rawat and Meena 2014). Opinions differ on the extent to which this happens (Abbott et al. 2010). If professors then also have little time for teaching because they must focus on producing more publications, the quality of academic education suffers (Rawat and Meena 2014; Miller et al. 2011).

## Getting rid of the fixation on publication metrics is crucial in the fight against fake papers

To sum up, there are various reasons why the scientific community needs to rethink its fixation on publication metrics when it comes to filling professor positions in academia and awarding research grants. In a recent study, using leading German pharmacologists as a case study, it has been shown that bibliometric parameters are arbitrary and can be interpreted (and hence, manipulated) one way or the other (Fox and Seifert 2024). If one just chooses the “right” bibliometric parameter, even a “bottom” scientist can be propelled to the “top,” and a “top” scientist can be sent to the “bottom.” Eliminating the mantra of bibliometric parameters as a proxy of perceived scientific excellence in academia is probably the best long-term solution to the fake paper problem, as it eliminates the reason for their publication and thus avoids an arms race between the methods of forgery and the methods of detection. In medicine, one would speak of treating causally instead of just symptomatically. Fortunately, there is an awareness of problematic standards in the assessment of research. The Declaration on Research Assessment (DORA) (https://sfdora.org/) is a statement and initiative advocating better approaches to research assessment. The statement has now been signed by over 25,000 individuals and organizations. Establishing such structural changes will of course take time. It will be crucial to instill the mindset in the education of the next generation of scientists that truthful scientific findings are the highest value, not metrics.

## Fighting fake papers is the responsibility of the entire scientific community

Appropriate education of scientists by universities and research institutions would not only help against fake papers. According to Tang (2024), there are also unintentionally questionable research practices due to a lack of knowledge of good scientific practice. Better education of scientists in good scientific practice and the acquisition of truthful knowledge would therefore also increase the quality of scientific publications whose authors have not intentionally fabricated data, but use questionable practices such as p-hacking or selective reporting to be able to tell a supposedly “convincing story.” Uneducated authors may assume that journal editors like such “convincing stories” to increase the acceptance probability of the paper. Rather, *Naunyn-Schmiedebergs Arch Pharmacol* accepts even “incomplete” stories, provided that the limitations of the study are discussed and future directions of research are properly outlined, e.g. with a scheme highlighting open questions. With this policy, we hopefully reduce the pressure of authors to create a “convincing story.” Reporting of true data has priority in our journal.

Authors must ensure that their work complies with good scientific practice at all times. Institutions should promote and monitor compliance with good scientific practice. The journals are responsible for thoroughly reviewing papers before they are published. In case of reasonable suspicion of scientific misconduct in an already published paper, they must investigate this conscientiously and transparently. Finally, readers can participate in post-publication reviews. Fighting fake papers and promoting truthful research is thus the responsibility of the entire scientific community.

## Unfortunately, truthful research on fake papers will be needed

Since fake papers will remain a major problem at least in the intermediate future, further research into how they can be identified is urgently needed. Such “truthful fake paper research” needs to be conducted with large datasets so that the findings on this topic are no longer based on assumptions, anecdotal evidence, and small sample sizes. The original research papers listed in Table 1 provide a solid methodological and conceptual starting point for “truthful fake paper research.” *Naunyn-Schmiedebergs Arch Pharmacol* has become a target of paper mills and is committed to fighting them and publishing original papers and reviews on research integrity and fake paper research. Dishonest paper mill authors publishing in *Naunyn-Schmiedebers Arch Pharmacol* should know that we will follow up on every single case of suspected fake science and that such papers will not prevail in the long run. New software tools will support our efforts in due course.

## Data Availability

No datasets were generated or analyzed during the current study.
